# CHIR99021 and Brdu Are Critical in Chicken iPSC Reprogramming via Small-Molecule Screening

**DOI:** 10.3390/genes15091206

**Published:** 2024-09-13

**Authors:** Kai Jin, Jing Zhou, Gaoyuan Wu, Zeyu Li, Xilin Zhu, Youchen Liang, Tingting Li, Guohong Chen, Qisheng Zuo, Yingjie Niu, Jiuzhou Song, Wei Han

**Affiliations:** 1 Joint International Research Laboratory of Agriculture and Agri-Product Safety of Ministry of Education of China, Yangzhou University, Yangzhou 225009, China; zhoujing980111@163.com (J.Z.); mz120221506@stu.yzu.edu.cn (G.W.); mx120230901@stu.yzu.edu.cn (Z.L.); mz120231564@stu.yzu.edu.en (X.Z.); adam3042705712@outlook.com (Y.L.); ltt_10356@126.com (T.L.); ghchen2019@yzu.edu.cn (G.C.); 006664@yzu.edu.cn (Q.Z.); 007510@yzu.edu.cn (Y.N.); 2 Key Laboratory of Animal Breeding Reproduction and Molecular Design for Jiangsu Province, College of Animal Science and Technology, Yangzhou University, Yangzhou 225009, China; 3 Institutes of Agricultural Science and Technology Development, Yangzhou University, Yangzhou 225009, China; 4 College of Bioscience and Biotechnology, Yangzhou University, Yangzhou 225009, China; 5 Department of Animal & Avian Sciences, University of Maryland, College Park, MD 20742, USA; songj88@umd.edu; 6 Jiangsu Institute of Poultry Sciences/Poultry Institute, Chinese Academy of Agricultural Sciences, Yangzhou 225125, China; hanwei830@163.com

**Keywords:** CHIR99021, BrdU, chicken iPSC, small molecule, reprograming

## Abstract

**Background/Objectives:** Induced pluripotent stem cells (iPSCs) reprogrammed from somatic cells into cells with most of the ESC (embryonic stem cell) characteristics show promise toward solving ethical problems currently facing stem cell research and eventually yield clinical grade pluripotent stem cells for therapies and regenerative medicine. In recent years, an increasing body of research suggests that the chemical induction of pluripotency (CIP) method can yield iPSCs in vitro, yet its application in avian species remains unreported. **Methods:** Herein, we successfully obtained stably growing chicken embryonic fibroblasts (CEFs) using the tissue block adherence method and employed 12 small-molecule compounds to induce chicken iPSC formation. **Results:** The final optimized iPSC induction system was bFGF (10 ng/mL), CHIR99021 (3 μM), RepSox (5 μM), DZNep (0.05 μM), BrdU (10 μM), BMP4 (10 ng/mL), vitamin C (50 μg/mL), EPZ-5676 (5 μM), and VPA (0.1 mM). Optimization of the induction system revealed that the highest number of clones was induced with 8 × 10^4^ cells per well and at 1.5 times the original concentration. Upon characterization, these clones exhibited iPSC characteristics, leading to the development of a stable compound combination for iPSC generation in chickens. Concurrently, employing a deletion strategy to investigate the functionality of small-molecule compounds during induction, we identified CHIR99021 and BrdU as critical factors for inducing chicken iPSC formation. **Conclusions:** In conclusion, this study provides a reference method for utilizing small-molecule combinations in avian species to reprogram cells and establish a network of cell fate determination mechanisms.

## 1. Introduction

Embryonic stem cells (ESCs) have the ability to generate all cell types in the body, though they are classified as pluripotent rather than totipotent, as no complete organism has been produced using ESCs alone [1]. ESCs can serve as valuable models for studying cell differentiation, fate determination, and the biochemical analysis of transcription factors. In recent years, stem cell research has become increasingly influential in biology and medicine, with ESCs poised to play a central role in biomedical research. They are key to regenerative medicine—considered the third therapeutic approach after drug therapy and surgery—and are also ideal for studying signal transduction, development, and epigenetics [2]. Lastly, ESCs hold significant potential as valuable tools for drug screening and safety assessments [3,4]. Despite the excitement surrounding stem cell research, we are still in the early stages of understanding the molecular mechanisms of stem cells in normal development, disease, and regeneration. However, the unavailability of expandable sources in large numbers of primary cells is a critical limitation. However, experimentally, a major problem with this kind of application is that ESCs are challenging to obtain, few in number, and problematic for routine large-scale culture and clonal selection. What is more, utilization of ESCs has faced the problem of immune rejection and various ethical issues for many years. These factors limit their application in pharmaceutics and the life sciences. Therefore, an alternative to ESCs could be considered a promising source for cell therapy.

Induced pluripotent stem cell (iPSC) reprogramming allows differentiated somatic cells to become pluripotent, similar to embryonic stem cells (ESCs), with the ability to differentiate into any somatic cell type and replicate indefinitely [5,6]. Both iPSCs and ESCs express pluripotency factors and surface markers and can form the three germ layers. iPSCs, however, have advantages over ESCs: they are readily available, avoid ethical concerns, and are less likely to cause immune rejection, making them ideal for patient-specific therapies, regenerative medicine, and drug development [7]. In 2006, Takahashi and Yamanaka demonstrated that somatic cells in mice could be reprogrammed into iPSCs using four transcription factors—Oct4, Klf4, Sox2, and c-Myc (known as Yamanaka factors) [8]. A year later, human iPSCs were successfully created [5,6]. This breakthrough shifted how we study developmental processes and disease models and opened new possibilities in regenerative medicine. However, the use of viral delivery and oncogenes in iPSC creation raises safety concerns for therapeutic use, prompting ongoing research into small-molecule substitutes for these reprogramming factors [9,10]. Although both ESCs and iPSCs have been used to model human genetic diseases, iPSCs have become the preferred option due to their accessibility and the lack of ethical issues. iPSCs may retain some epigenetic memory from their original somatic cells, which could affect their differentiation potential, but both iPSCs and ESCs have shown similar effectiveness in disease modeling.

Further improvements in reprogramming factors have been demonstrated in various mammalian studies. To make iPSCs safer for clinical use, several non-integrating methods were developed to avoid the risks of insertional mutagenesis and genetic alterations associated with viral methods [11]. These include reprogramming using episomal DNAs [12,13], adenoviruses [14], Sendai mRNAs [15], microRNAs [16,17], and small molecules [18]. Since *Oct4*, *Sox2*, *Myc*, and *Klf4* regulate key signaling pathways, small molecules targeting these pathways have emerged as alternatives to genetic reprogramming [19,20]. Compared to genetic methods, small molecules offer several advantages: they are easier to use, provide more precise control, and can be adjusted by changing concentrations. Therefore, the chemical approach, or chemical induction of pluripotency (CIP), has been shown to enhance iPSC generation and differentiation. Notably, Deng and colleagues replaced *Oct4* with the small molecule Forskolin, creating a protocol for generating chemically induced pluripotent stem cells (CiPSCs) [18]. Many studies also have demonstrated that small molecules can replace Yamanaka factors (OSKM). For example, Vitamin C can replace *Myc*, RepSox can substitute for Sox2 [21], and BrdU can replace Oct4 [22]. Various compounds like VPA, CHIR99021, RepSox, and Forskolin can induce iPSC formation, with some achieving the same effects as Yamanaka factors. Small molecules are not only useful for generating specific cell types in vitro for disease modeling and transplantation but also hold promise for drug development to stimulate cell repair and regeneration in patients. In 2018, Duanqing Pei’s team optimized the reprogramming process using a combination of 12 compounds, including CHIR99021, Vitamin C, BMP4, and others, creating a serum-free culture medium that improved the stability and efficiency of iPSC cloning [23].

As of present, the induced pluripotent stem cell (iPSC) induction technique has been widely applied in various species including mice [8], rats [24], monkeys [25], pigs [26], sheep [27], cattle [28], horses [29], and humans [6], but its application in poultry species is less reported. In poultry, research on iPSC induction started relatively late. In 2012, Lu [30] obtained quail iPSCs (qiPSCs) by infecting quail with lentiviruses carrying six human stem cell genes: *POU5F1*, *NANOG*, *SOX2*, *LIN28*, *KLF4*, and *C-MYC*. In 2017, Kim [31] used a calcium phosphate co-precipitation method to prepare retroviral vectors pVSV-G, pMXs-Oct3/4, pMXs-Sox2, pMXs-klf4, pMXs-cMyc, and pMXs-Nanog, inducing feather follicle cells (FFCs) to generate induced pluripotent stem-like cells (iPSLCs) in poultry. However, to date, there have been no reports on the induction of chicken iPSCs using small-molecule compounds. In order to further explore the optimal induction pathway for obtaining pluripotent stem cells, this experiment aims to induce the formation of chicken iPSCs using different small-molecule compounds and to screen for key combinations of small-molecule compounds, providing cellular materials for the application of embryonic stem cells (iPSCs).

## 2. Materials and Methods

### 2.1. Animal Ethics Approval

The experiment used freshly fertilized eggs of black-boned silky fowl, approved by the Animal Care and Use Committee of Yangzhou University (permit number: SYXK [Su] IACUC 2012-0029). All experimental procedures were conducted in strict accordance with the regulations outlined in the “Regulations for the Administration of Experimental Animals” and approved by the State Council of the People’s Republic of China.

### 2.2. Isolation and Culture of CEFs

The embryos (Rugao Yellow Chicken, from Jiangsu Institute of Poultry Sciences) at embryonic days 9–11 (HH 35-37) were retrieved and disinfected using 0.1% benzalkonium bromide solution (Nanchang Baiyun Pharmaceutical Co., Ltd., Nanchang, China, H36021593) and 75% alcohol. After disinfection, the blunt end of the eggs was carefully cracked open using ophthalmic forceps, and the embryos were removed, with their heads, limbs, and internal organs removed. Subsequently, the muscle tissue devoid of bones was cut into tissue blocks measuring 1 mm × 1 mm × 1 mm. These tissue blocks were then placed in a 24-well plate, and a drop of serum was added. After fixation by 4% paraformaldehyde (Nanchang Baiyun Pharmaceutical Co., Ltd., Nanchang, China, P0099) for half an hour, DMEM high-glucose culture medium containing 10% FBS (Gibco, New York, NY, USA, 10099141) was slowly added along the well wall. Upon the release of a significant number of cells from the tissue blocks, passaging was performed. During passaging, cells were washed twice with PBS, digested with trypsin (Gibco, New York, NY, USA, 25200072) for 3 min, and the digestion was terminated with an equal amount of complete DMEM medium. After centrifugation, cells were thoroughly resuspended in complete medium, reseeded in wells, and placed in a 37 °C, 5% CO_2_ incubator. It is recommended to use CEFs within the F7 generation for iPSC induction, with the best results obtained from CEFs in the F2–F3 generations.

### 2.3. CCK-8 Cell Proliferation Assay

A portion of the cells was counted using a cell counter and evenly seeded into a 96-well plate for proliferation culture (approximately 5000 cells/well). Then, 10 μL of CCK-8 solution (Vazyme, Nanjing, China, A311) was added along the walls of the cell plate wells. After gently mixing, the plate was placed in a 37 °C cell culture incubator in the dark and incubated. Absorbance values at 450 nm were measured using a microplate reader.

### 2.4. iPS Induction

CEFs prepared for the feeding layer were treated with thymidine C (MCE, Monmouth Junction, NJ, USA, HY-13316). Subsequently, CEFs obtained via the tissue block attachment method were seeded onto the feeding layer for induction. The iPSC induction culture medium consisted of 43.5 mL knockout DMEM medium (Gibco, New York, NY, USA, 10829018) supplemented with 20 μL of β-mercaptoethanol (0.1 mmol/L, Sigma, St. Louis, MO, USA, M3148), 200 μL of MEM non-essential amino acid solution (NEAA) (Gibco, 11140050), 100 μL of SCF (5 ng/mL, Sigma, St. Louis, MO, USA, SRP3151), 100 μL of bFGF (10 ng/mL, Sigma, St. Louis, MO, USA, F0291), 50 μL of LIF (1 ng/mL, Millipore, Burlington, MA, USA, ESG1106), 1 mL of chicken serum (Gibco, NY, USA, 16110082), 500 μL of penicillin–streptomycin (Solarbio, Beijing, China, P1400), KSR (Gibco, New York, NY, USA, 10828010), and corresponding concentrations of the small-molecule compounds RepSox (MCE, Monmouth Junction, NJ, USA, HY-13012), CHIR99021 (MCE, Monmouth Junction, NJ, USA, HY-10182), DZNep (MCE, Monmouth Junction, NJ, USA, HY-12186), Forskolin (MCE, Monmouth Junction, NJ, USA, HY-15371), VPA (MCE, Monmouth Junction, NJ, USA, HY-10585), Vitamin C (MCE, Monmouth Junction, NJ, USA, HY-B0166), BrdU (MCE, Monmouth Junction, NJ, USA, HY-15910), SGC0946 (MCE, Monmouth Junction, NJ, USA, HY-15650), BMP4 (MCE, Monmouth Junction, NJ, USA, HY-P7007), bFGF (MCE, Monmouth Junction, NJ, USA, HY-P7331), AM580 (MCE, Monmouth Junction, NJ, USA, HY-10475), and EPZ-5676 (MCE, Monmouth Junction, NJ, USA, HY-15593) for induction (Table 1).

### 2.5. Quantitative Real-Time Polymerase Chain Reaction

RNA was extracted from the cells using TRIzol (Tiangen, Beijing, China, DP424), and its concentration was measured. Reverse transcription was carried out with HiScript III RT SuperMix for qPCR (+gDNA wiper) (Vazyme, Nanjing, China, R323-01). The resulting cDNA was used with ChamQ Universal SYBR qPCR Master Mix (Vazyme, Nanjing, China, Q711-02) to assess the mRNA expression levels of pluripotency genes, including *Gata6*, *Lin28A*, *Nanog*, *Oct4*, *Sall4*, *Sox2*, and *SSEA-1*. The *β-actin* gene served as the internal reference, and data analysis was performed using the 2^−ΔΔCt^ method as described [8]. The qRT-PCR primers are listed in Table 1.

### 2.6. Immunofluorescence Staining

The collected cells were washed once with PBS buffer, fixed with 4% paraformaldehyde at room temperature for 30 min, rinsed with PBS to remove the fixative, permeabilized with 1% Triton-X100 (Solarbio, Beijing, China, T8200) at room temperature for 15 min, followed by another wash with PBS. The samples were then blocked with PBS containing 10% FBS for 2 h, followed by overnight incubation with SSEA-1 antibody (Biotechne, Minneapolis, MN, USA, IC2155T, 1:1000). The next day, the samples were washed with PBS containing 0.1% Tween-20 (Solarbio, Beijing, China, T8220) and the cell nuclei were stained with 5 ng/µL DAPI (Beyotime, Beijing, China, C1002). Image acquisition was performed using a confocal microscope (Olympus, Tokyo, Japan, FV1200). CEFs served as negative controls.

### 2.7. Flow Cytometric Analysis

The cells were collected in a 1.5 mL centrifuge tube and washed with PBS buffer. After washing, 1% Triton-X100 was added and incubated at room temperature for 15 min to permeabilize the cells. After centrifugation, the cells were washed again with PBS. Subsequently, the cells were blocked with 10% FBS-PBS for 2 h, followed by complete removal of the supernatant after centrifugation. SSEA-1 antibody was then used to label the protein overnight. The next day, the cells were washed with PBST and resuspended in PBS for analysis and detection of staining signals using FACS LSRFortessa (BD Biosciences, San Jose, CA, USA). CEFs served as negative controls. The gating strategy using the FSC-A (forward scatter area) vs. FSC-W (forward scatter width) plot was used to identify and gate single cells. Doublets and clumps typically have different FSC-W values compared to single cells.

### 2.8. Alkaline Phosphatase

The cells were collected, and their alkaline phosphatase (ALP) activity was assessed using an alkaline phosphatase assay kit (Solarbio, Beijing, China, G1480). After washing the cells with PBS, they were treated with ALP fixing solution for 3 min. Subsequently, distilled water was gently added to wash the cells for 5–10 s. Then, the cells were incubated in the prepared ALP incubation solution in a light-protected humidified chamber for 15–20 min. After washing with PBS, the cells were counterstained with Nuclear Fast Red Staining solution for 3–5 min. Cell images were captured using a fluorescence inverted microscope (Olympus, Japan, Tokyo, IX51).

### 2.9. EdU Cell Proliferation Assay

The cells in logarithmic growth phase were seeded into a 96-well plate for cultivation and labeled using the Cell-LightTM EdU Apollo In Vitro Kit (Ruibo, Beijing, China, C10310-1). After washing the cells with PBS, they were fixed with 4% paraformaldehyde for 30 min, followed by a 5 min incubation with glycine on a shaker to quench the fixative. Subsequently, the cells were washed again with PBS and then incubated with permeabilization buffer (PBS containing 0.5% Triton X-100) on a shaker for 10 min. After another round of PBS washing, the cells were stained in the dark for 30 min using 1× Apollo^®^ staining reaction mixture. Following this, the cell nuclei were stained with 1 mg/mL Hoechst 33,342 (Invitrogen, Carlsbad, CA, USA, R37165) for 3–5 min. After two washes with permeabilization buffer, the cells were mounted with anti-fade mounting medium and observed under a confocal microscope.

### 2.10. Statistical Analysis

Statistical analysis was performed using SPSS software (Ver. 19.0). A one-way ANOVA was used, with * *p* < 0.05 indicating significance, ** *p* < 0.01 indicating high significance, and *** *p* < 0.001 indicating very high significance. Tukey’s post hoc test was used for multiple comparisons. All experiments were repeated at least three times. Graphs were created with GraphPad Prism Ver. 7.0 (GraphPad Software, Dotmatics, https://www.graphpad.com/ (accessed on 10 August 2023)), and flow cytometry images were analyzed with FlowJo Ver. 10.0 (Becton, Dickinson & Company, https://www.flowjo.com/ (accessed on 10 August 2023)).

## 3. Results

### 3.1. Stably Produced CEFs Can Be Obtained by the Tissue Block Culture Method

**Figure 1:**
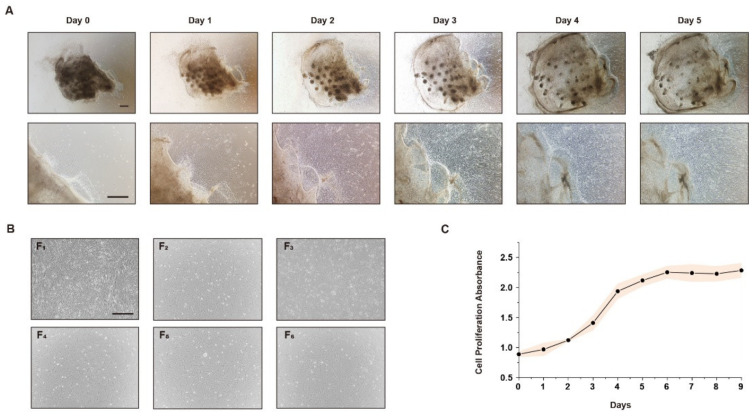
(**A**): CEFs obtained from fresh tissue block migration morphology (200×). (**B**): Morphological observation of CEFs after passage (200×). (**C**): CCK-8 assay to detect the proliferation rate of CEFs.

To obtain a large number of available CEFs for iPSC reprogramming, muscle tissue was collected from chicken embryos at 9–11 days (HH St.35-37) to isolate CEFs by the tissue block culture method. The results show that a small number of cells dissociated from the surrounding tissue blocks after 1 day of culture. Cells migrated out of most tissue blocks, and the number of CEFs increased rapidly when cultured to days 2–3. On day 5, the CEFs were confluent to the 24-well plate and could be passaged (Figure 1A). According to the cell morphological observation, the morphology of CEFs passed to the 6th passage did not change significantly and still maintained the typical characteristics of fibroblasts (Figure 1B). To ensure that the passaged cells could be used for subsequent experiments, the proliferation ability of the isolated cells was tested by the CCK-8 assay and growth curves were plotted. The results show that the cells proliferated well and the proliferation curve was S-shaped (Figure 1C). The above results indicate that CEFs isolated by the tissue block culture method can be stably passaged and used for further studies.

### 3.2. Establishment of Chicken iPSC Formation Induced by Small-Molecule Compounds

Studies have shown that the formation of iPSCs involves a variety of biological processes such as the expression of endogenous pluripotency genes, the activation and inhibition of signaling pathways, and the reprogramming of epigenetic modifications. According to this characteristic, we designed concentration gradients based on the minimum lethal dose of each small-molecule compound and finally determined the composition of small-molecule compounds (Table 2). To explore the optimal induction system and culture conditions of iPSCs, CEFs with different cell densities were inoculated with different small-molecule combinations to induce iPSCs and observe the induction efficiency.

**Figure 2:**
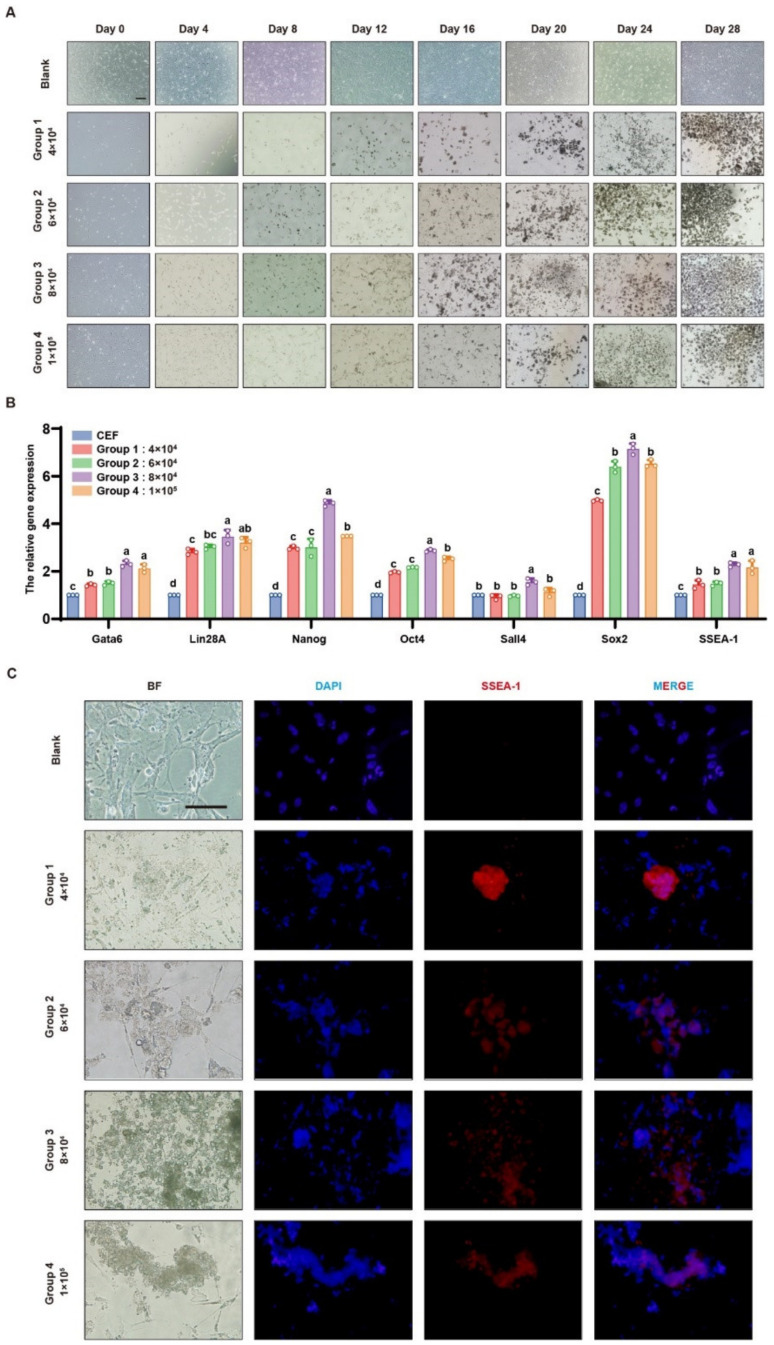
(**A**): Morphological observation (200×) of iPSC formation induced from CEFs at different densities; (**B**): qRT-PCR detection of the expression levels of cell-related genes during iPSC induction process. Different lower-case letters indicate a significant difference among different gene expression levels (*p* < 0.05); (**C**): Indirect immunofluorescence detection of SSEA-1 protein expression in iPSCs (400×).

The optimal initial cell seeding density was determined by performing non-passaged induction at four different densities: 4 × 10^4^, 6 × 10^4^, 8 × 10^4^, and 1 × 10^5^ cells/well. Based on cell morphology and immunofluorescence results, 8 × 10^4^ cells/well was identified as the best density, as cells underwent noticeable morphological changes during induction, ultimately forming iPSC colonies by day 18 (Figure 2A,C). Quantitative real-time PCR (qRT-PCR) further confirmed that the 8 × 10^4^ cells/well group had the highest expression levels of pluripotency genes, including Gata6, Lin28A, Nanog, Oct4, Sall4, Sox2, and SSEA-1, all of which were significantly higher than in the other groups (*p* < 0.05) (Figure 2B). Thus, 8 × 10^4^ cells/well was selected as the optimal seeding density for iPSC induction.

**Figure 3:**
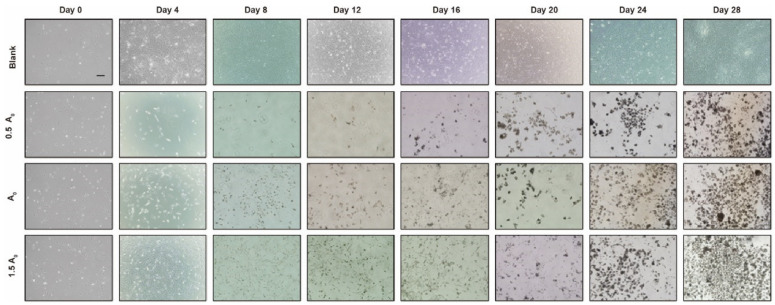
Morphological observation (200×) of iPSC formation induced from CEFs in different concentration treatment groups.

To confirm the effect of small-molecule compound concentration on induction efficiency, the experiment was performed with the above optimal cell density of 8 × 10^4^ cells/well, and the initial concentrations of induction combinations were set as group A0, namely: bFGF (10 ng/mL) + CHIR99021 (3 μM) + RepSox (5 μM) + Forskolin (10 μM) + DZNep (0.05 μM) + BrdU (10 μM) + AM580 (0.05 μM) + BMP4 (10 ng/mL) + Vitamin C (50 μg/mL) + EPZ-5676 (5 μM) + SGC0946 (5 μM) + VPA (0.1 μM). Based on the small-molecule compound combination of group A0, group A1 was designed with a concentration of 0.5 times group A0, and group A2 was designed with a concentration of 1.5 times group A0. The results demonstrate that among the three induced groups, the most round dense cell clusters appeared in group A2, followed by group A0, and finally group A1 (Figure 3). This result indicates that 1.5 times the A0 concentration is the most suitable for inducing the reprogramming of CEFs into iPSCs, which is the optimal induction concentration, namely: group A2 = bFGF (15 ng/mL) + CHIR99021 (4.5 μM) + RepSox (7.5 μM) + Forskolin (15 μM) + DZNep (0.075 μM) + BrdU (15 μM) + AM580 (0.075 μM) + BMP4 (15 ng/mL) + Vitamin C (75 μg/mL) + EPZ-5676 (7.5 μM) + SGC0946 (7.5 μM) + VPA (0.15 mM) had the best induction efficiency.

### 3.3. iPSCs Induced by Small-Molecule Compounds Show Typical Biological Characteristics of ESCs

**Figure 4:**
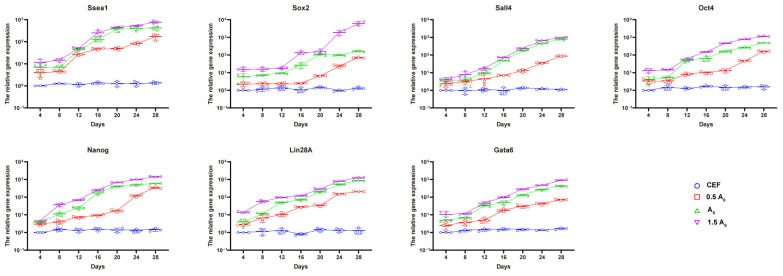
qRT-PCR of the expression levels of related genes during the induction process of iPSCs.

To clarify the biological characteristics of the induced iPSCs, we collected iPSCs induced at different concentrations for 4 d, 8 d, 12 d, 16 d, 20 d, 24 d, and 28 d and detected the expression of the pluripotency marker genes *Gata6*, *Lin28A*, *Nanog*, *Oct4*, *Sall4*, *Sox2*, and *SSEA-1* in each group by qRT-PCR. The results determine that the expression levels of all pluripotency genes in group A2 were significantly higher than those in groups A0 and A1 (*p* < 0.05), and the expression reached the highest level on day 28 (Figure 4), which further proved that group A2 was the most suitable concentration for inducing iPSC formation.

**Figure 5:**
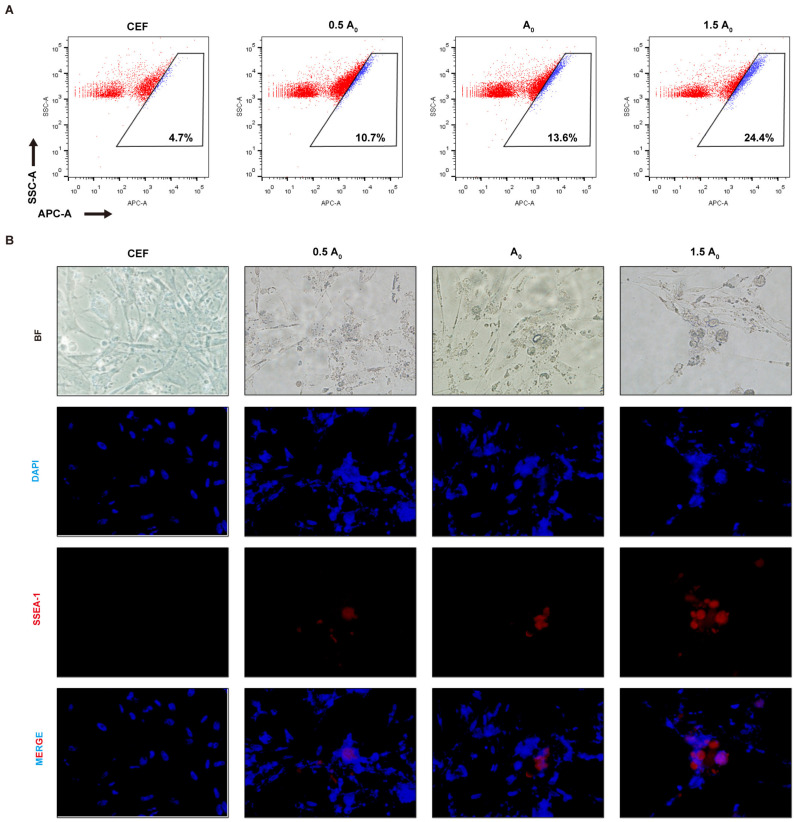
(**A**): Flow cytometry analysis of the percentage of SSEA-1-positive cells. (**B**): Immunofluorescence of SSEA-1 expression (400×).

**Figure 6:**
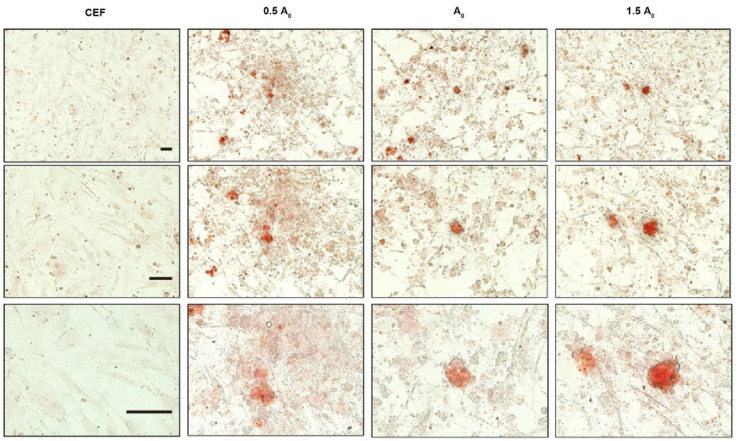
Identification of alkaline phosphatase activity in iPSC clones (200×).

**Figure 7:**
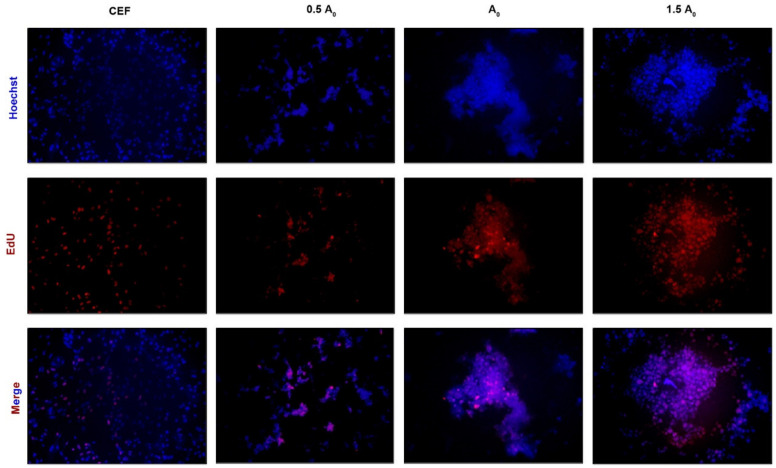
EdU staining in iPSC clones (400×).

In this study, flow cytometry analysis was performed on iPSCs induced up to day 28 in different groups using the pluripotency marker protein antibody SSEA-1. The results show that the expression of the SSEA-1 protein was highest in group A2 (24.4%), followed by group A0 (13.6%) and finally group A1 (10.7%) (Figure 5A). Similar results were also obtained by immunofluorescence (Figure 5B). All these results not only indicate that group A2 was the optimal concentration for inducing iPSC formation, but also confirm that induced iPSCs had similar biological properties to ESCs and could express pluripotent genes or proteins. To further validate the conclusion, we performed AKP staining on iPSCs from all groups. The results show that group A2 exhibited more stainable cells, indicating that it induced more pluripotent cells, followed by group A0 and finally group A1 (Figure 6). Moreover, the EdU cell proliferation assay was used to test the proliferation and passaging ability of iPSCs in vitro. According to fluorescence microscope observation, iPSCs formed in group A2 had strong proliferation ability, and the cells grew well after passaging, which could be used for subsequent experimental studies (Figure 7). 

Based on the results above, we combined group A2 as follows: (bFGF (15 ng/mL) + CHIR99021 (4.5 μM) + RepSox (7.5 μM) + Forskolin (15 μM) + DZNep (0.075 μM) + BrdU (15 μM) + AM580 (0.075 μM) + BMP4 (15 ng/mL) + Vitamin C (75 μg/mL) + EPZ-5676 (7.5 μM) + SGC0946 (7.5 μM) + VPA (0.15 mM)) and concluded that it had the best induction efficiency, which could be used as the initial system for the induction reprogramming of CEFs into iPSCs for subsequent induction system optimization experiments, with the aim of improving the induction efficiency and shortening the induction period.

### 3.4. CHIR99021 and BrdU Are Small-Molecule Compounds Necessary for the In Vitro Induction of Reprogramming in iPGCs

**Figure 8:**
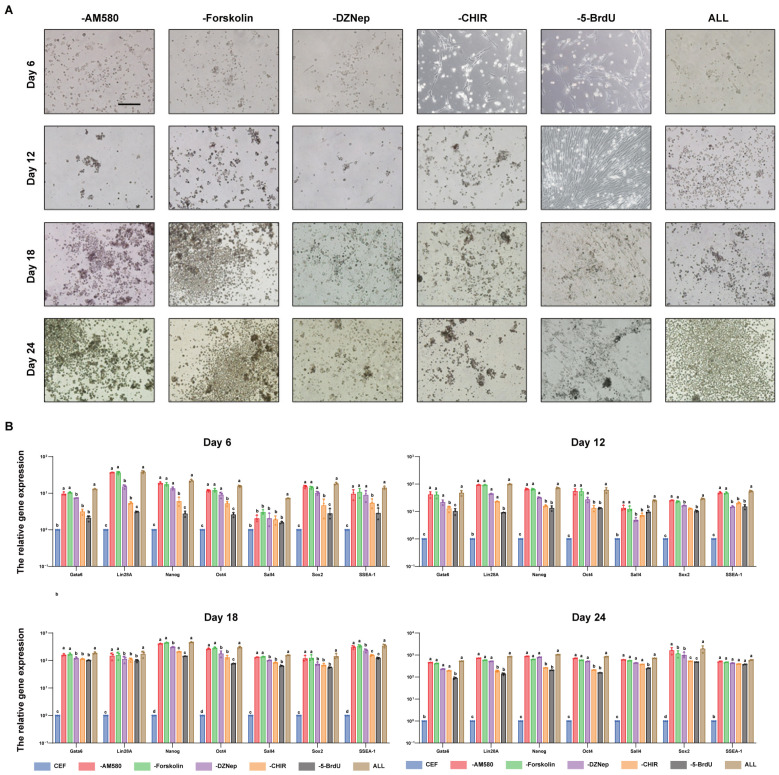
(**A**): Cellular morphological observation of iPSC formation induced by different small-molecule combinations (200×). (**B**): qRT-PCR analysis of the expression of cell-related genes during the induction of iPSCs. Different lower-case letters indicate a significant difference among different gene expression levels (*p* < 0.05).

To seek the best combination of small-molecule compounds, compounds were deleted one by one in this study. The results of iPSC cell morphology observation show that compared with the initial system induction group, the morphological changes in cells in the CHIR99021- and BrdU-deleted groups were not apparent in the early stage of induction (day 6), and then the dense cell clusters appeared with round shapes on days 12 and 18 of induction. In contrast, in the groups lacking AM580, Forskolin, and DZNep, the starting time and trend of pluripotency gene expression were the same as those in the initial system induction group. In addition, these groups showed significant changes in cell morphology on day 6 of induction, round dense cell clusters on day 12, and a notable increase in cell mass on day 18. This suggests that CHIR99021 and BrdU were key small molecules for the induction of iPSC formation, while AM580, Forskolin, and DZNep had little effect on the induced state. In addition, to explore the combination of small-molecule compounds with the best effect, we used qRT-PCR to detect the expression of pluripotency genes. The results show that the expression of the pluripotency genes *Gata6*, *Lin28A*, *Nanog*, *Oct4*, *Sall4*, *Sox2*, and *SSEA-1* was lower and the expression time was later after the deletion of BrdU and CHIR99021. However, there was no significant difference in gene expression after the removal of AM580, Forskolin, and DZNep (Figure 8A,B).

The results mentioned above indicate that the deletion of BrdU and CHIR99021 can slow down the process of cell induction, thus BrdU and CHIR99021 played an essential role in the process of chicken CEF-induced reprogramming into iPSCs, while the effects of AM580, Forskolin, and DZNep, were weaker than those of the former two. In summary, the results of our study confirm that CHIR99021 and BrdU were the important small-molecule compounds in chicken-induced reprogramming.

## 4. Discussion

The reprogramming of somatic cells using defined factors, which enables the conversion of cells from one lineage to another, has significantly transformed our understanding of cell identity and fate determination. Following the discovery of iPSCs, it became possible to regenerate pluripotent states from various somatic cell types, leveraging insights from developmental biology. While reprogramming has been successfully achieved both in vitro and in vivo, several challenges must still be addressed before this approach can be widely applied in clinical settings.

In 2006 and 2007, Yamanaka and colleagues successfully reprogrammed mouse and human somatic cells into iPSCs by ectopically expressing four transcription factors (*Oct4*, *Sox2*, *Klf4*, and *cMyc*) [5,8]. However, this genetic approach raised safety concerns due to the use of viral delivery systems and the permanent integration of exogenous oncogenes, limiting its therapeutic potential. A major issue with both hiPSC and hESC transplantation is the risk of teratoma formation. Even a small number of residual pluripotent stem cells (PSCs) can lead to teratomas. Additionally, lineage-specific stem cells within a transplant may form tumors if incorrectly or incompletely patterned. For example, neural stem cells improperly directed toward cortical development can form proliferative structures like “neural rosettes”, which may grow in a tumor-like manner if transplanted in vivo [18]. As a result, researchers have devoted significant effort to finding ways to prevent teratomas and other tumors caused by improper cell patterning.

Numerous studies have explored strategies to improve reprogramming efficiency and reduce tumorigenicity. These include using alternative transcription factors [9], chemical inhibitors [10], genetic factors [11], microRNAs (miRNAs) [12], and signaling molecules [13]. Small non-coding RNAs, in particular, play a key role in regulating reprogramming by enhancing iPSC generation and differentiation [14]. Researchers are no longer content with merely inducing the production of iPSCs but are instead continuously seeking a safer and more effective induction pathway. The aim is to minimize the potential contamination and carcinogenicity during the iPSC formation process while ensuring low risk and to obtain cells with higher efficiency, purity, and quality, with the ultimate goal of widespread clinical application.

The initial establishment of iPSC induction systems involved the overexpression of OSKM mediated by retroviral vectors. But the retroviruses as carriers for introducing exogenous genes into somatic cells face the risk of viral fragment insertion into the genome, leading to cellular gene mutations and an increased risk of tumor carcinogenesis. Additionally, the inclusion of factors such as *Klf4* and *c-Myc* also carries potential oncogenicity, posing a risk of inducing carcinogenesis.

All four reprogramming factors have been linked to tumorigenicity, especially *c-Myc*, one of the most frequently mutated genes in human cancers and a common driver mutation. In fact, studies have shown that chimeric mice created with iPSCs generated through retroviral transfection of these factors often develop tumors [9]. Another concern is whether the reprogramming process itself causes mutations [32]. Gore et al. performed whole-exome sequencing on human iPSC lines, finding around 10 novel non-synonymous SNVs per iPSC clones, suggesting that iPSC reprogramming may be mutagenic—a finding supported by other studies [33].

We detected reactivation of the *c-Myc* retrovirus in these tumors, while chimeric mice generated from iPSCs without *c-Myc* did not develop tumors. Although iPSCs can be reliably produced, the process remains inefficient, with fewer than 1% of transfected fibroblasts becoming iPSCs. This initially led to the hypothesis that iPSCs might originate from rare stem cells or undifferentiated cells present in fibroblast cultures. Interestingly, the low efficiency of establishing stable pluripotent cell lines, typically between 0.001% and 1% in mice, has prompted researchers to explore alternative methods. These include reducing the risks associated with oncogenic reprogramming factors, enhancing reprogramming efficiency, and improving iPSC cell quality [34]. Some studies have shown that Yamanaka factors (YFs) can be replaced by other genes and chemicals [35]. For example, *GATA3*, *GATA6*, *SOX7*, *PAX1*, and others have been found to promote reprogramming by replacing *Oct4* and *Sox2*. Using doxycycline with *GATA3*, *SOX2*, *KLF4*, and *c-MYC (G3SKM)* increased *Oct4* expression in mouse iPSCs by 20–40%. Additionally, inducing human iPSCs with *OCT4*, *SOX2*, *NANOG*, and *LIN28* generated 198 colonies from 900,000 IMR90 cells [6]. Our understanding of the molecular mechanisms underlying reprogramming has advanced significantly, leading to the discovery that small molecules can replace cytokines traditionally used to direct iPSC generation. Compared to introducing transcription factors (TFs) through viral methods, small molecules offer several advantages, such as reduced risks and greater control. A range of small molecules has been identified that can replace one or more TFs, enhancing reprogramming efficiency and speed.

For example, Huangfu et al. reported that valproic acid (VPA), a histone deacetylase inhibitor, enabled the reprogramming of human fibroblasts using only two factors, *Oct4* and *Sox2*, without the need for oncogenes like *c-Myc* or *Klf4* [36]. Additionally, chemical inhibition of TGF-β signaling with SB431542 increased reprogramming efficiency without *c-Myc* [21]. A major breakthrough came in 2013 when Hou et al. successfully reprogrammed mouse cells into iPSCs using a cocktail of seven small molecules, including VPA, CHIR99021, E616452, Tranylcypromine, Forskolin, DZNep, and TTNPB [18]. This all-chemical approach offered a 1000-fold increase in efficiency with further refinement using additional compounds like AM580, EPZ004777, and 5-aza-2-deoxycitidine [37]. Stadtfeld’s work showed that combining CHIR99021, a TGF-β antagonist (ALK5 inhibitor II), and Vitamin C could enhance reprogramming efficiency to 41%, while the addition of doxycycline increased this to 80% [34]. Similarly, Cao found that using a mix of Vitamin C, BMP4, RepSox, BrdU, and other small molecules resulted in 43% reprogramming efficiency in mouse fibroblasts. In our study, applying a similar combination of small molecules achieved a reprogramming efficiency of 24.4% in chicken somatic cells [23].

Chemically induced pluripotent stem cells (ciPSCs) displayed global gene expression profiles similar to ESCs, demonstrating that small molecules can reprogram cell fate without the need for ectopic expression of master regulator genes. This study provides proof of concept for an all-chemical reprogramming strategy, with potential applications in generating specific cell types for cell therapy. Most small molecules used to create ciPSCs fall into categories such as epigenetic modifiers, cell signaling and apoptosis regulators, Wnt signaling modulators, and metabolism or senescence modulators. Small-molecule modulators of key developmental pathways—such as Wnt, FGF, Hedgehog, Notch, and BMP/TGFβ—are crucial for hPSC differentiation.

Developmental signaling pathways like FGF, Wnt, Hedgehog, Notch, and TGFβ/BMP are critical for embryonic development and PSC differentiation both in vivo and in vitro. Small molecules that regulate these pathways are particularly valuable for guiding PSC differentiation into specific cell types. For instance, activating Wnt signaling through natural ligands [38] or chemically inhibiting glycogen synthase kinase 3 (GSK3) [39], a CTNNB1 (β-catenin) antagonist, has been shown to enhance iPSC formation. Additionally, small molecules that modulate chromatin-modifying enzymes can promote fibroblast reprogramming by facilitating chromatin remodeling, a key factor in iPSC generation. Therefore, uncovering the epigenetic mechanisms involved in iPSC formation and the critical cellular signaling pathways involved allows for the screening of chemical activators or inhibitors associated with chromatin modification and signaling pathways. This can impact the reprogramming efficiency and chromatin dynamics processes, thereby promoting iPSC formation.

The *GSK3* inhibitor CHIR99021, known to activate Wnt/β-catenin signaling and reduce *TCF3*’s repressive effect on pluripotency genes, has been shown to significantly boost reprogramming efficiency. *GSK3*, a serine/threonine kinase, regulates over 40 cellular proteins and plays a key role in the β-catenin/Wnt pathway, which is crucial for stem cell maintenance and proliferation [40]. Inhibiting *GSK3* enhances reprogramming, as demonstrated by Silva et al., who found that blocking both *MEK* and *GSK3* (with PD0325901 and CHIR99021) promoted the conversion of “pre-iPSCs” into fully reprogrammed iPSCs. Moreover, CHIR99021, combined with Parnate, enhanced the reprogramming of human keratinocytes transduced with *Oct4* and *Klf4* into iPSCs [41]. When used with BIX-01294 and BayK 8644, it also enabled mouse embryonic fibroblasts transduced by *Oct4* and *Klf4* to reprogram into iPSCs [8]. CHIR-99021 can stably regulate downstream effectors such as *c-Myc*, *β-catenin*, and others, thereby inhibiting cell differentiation and promoting self-renewal to maintain pluripotency. Additionally, CHIR-99021 can modulate various signaling pathways including Wnt/β-catenin, TGF-β, Nodal, MAPK [42,43], and the expression of epigenetic regulators like *Dnmt3l*, significantly increasing the efficiency of somatic cell reprogramming [44]. BrdU, as a nucleoside analog, competes with thymidine during DNA replication, commonly used to detect cell proliferation. Furthermore, BrdU is associated with chromatin remodeling, capable of opening sites enriched with GATAs, KLFs, and SOXs and closing AP1-enriched sites [23]. During the induction of iPSCs, BrdU primarily acts in the early stages, reducing genomic methylation levels, enhancing transcriptional activation, thus replacing *Oct4*, and directly facilitating somatic cell reprogramming, favoring iPSC induction and making the reprogramming process simpler and more efficient [22].

## 5. Conclusions

This experiment involved dissociating tissue blocks to obtain chicken embryo fibroblasts (CEFs). Various culture densities and compound concentrations were tested to induce CEFs into iPSCs, with the goal of identifying the optimal small-molecule combination for induction. The resulting iPSCs exhibited pluripotency characteristics similar to those of ESCs. The final optimized induction system involved seeding at a density of 8 × 10^4^ cells/well and using a specific combination of small molecules. Additionally, a deletion approach pinpointed CHIR99021 and BrdU as key functional molecules in the iPSC formation process. This study provides a scientific basis for refining induction systems, enhancing efficiency, and reducing induction times.

## Figures and Tables

**Table 1:** Primers and fragment sizes for qRT-PCR identification.

Gene Name	Prime (5′ → 3′)	Size (bp)
*Gata6*	F: GAAGATGCACAACTCGGAGATCAG R: GAGCCGTTTGGCTTCGTCA	100
*Lin28A*	F: GGCGTCTTCTGCATTGGC R: TGGCGACCATGTGGCTGA	178
*Nanog*	F: TGGTTTCAGAACCAACGAATGAAG R: TGCACTGGTCACAGCCTGAAG	180
*Oct4*	F: ACCAGCATCGAGACCAACGTGA R: TTGCAGAACCAGACCCGGACAA	137
*Sall4*	F: TGGTTTCAGAACCAACGAATGAAG R: TGCACTGGTCACAGCCTGAAG	180
*Sox2*	F: AGGCTATGGGATGATGCAAG R: GTAGGTAGGCGATCCGTTCA	160
*SSEA-1*	F: ACCAGCATCGAGACCAACGTGA R: TTGCAGAACCAGACCCGGACA	117
*β-actin*	F: CAGCCATCTTTCTTGGGTAT R: CTGTGATCTCCTTCTGCATCC	169

**Table 2:** Initial concentration and concentration gradient of small-molecule compounds.

Compound	Function	Initial Concentration	Concentration Gradient
RepSox	TGFβR-1/ALK5 Inhibitor	5 μM	0–7.5 μM
CHIR99021	Selective Activator of Glycogen Synthase Kinase 3β	3 μM	0–4.5 μM
DZNep	EZH2 Inhibitor	0.05 μM	0–0.075 μM
Forskolin	Adenylyl Cyclase Activator	10 μM	0–15 μM
VPA	HDAC1 Inhibitor	0.1 mM	0–0.15 mM
Vitamin C	Histone Demethylase	50 μg/mL	0–75 μg/mL
5-BrdU	DOT1L Inhibitor	10 μM	0–15 μM
SGC0946	DOT1L Inhibitor	5 μM	0–7.5 μM
BMP4	BMP4 Signaling Activator	10 ng/mL	0–15 ng/mL
bFGF	Cell Proliferation Promoter	10 ng/mL	0–15 ng/mL
AM580	RARα-Selective Agonist	0.05 μM	0–0.075 μM
EPZ-5676	DOT1L Inhibitor	5 μM	0–7.5 μM

## Data Availability

The original contributions presented in the study are included in the article, further inquiries can be directed to the corresponding author.
